# Interpretations arising from Wrightian and Malthusian fitness under strong frequency dependent selection

**DOI:** 10.1002/ece3.500

**Published:** 2013-04-03

**Authors:** Bin Wu, Chaitanya S Gokhale, Matthijs van Veelen, Long Wang, Arne Traulsen

**Affiliations:** 1 Research Group for Evolutionary Theory, Max-Planck-Institute for Evolutionary BiologyAugust-Thienemann-Straße 2, 24306, Plön, Germany; 2 Center for Systems and Control, State Key Laboratory for Turbulence and Complex Systems, College of Engineering, Peking UniversityBeijing, 100871, China; 3 CREED, University of AmsterdamRoetersstraat 11, 1081, WB Amsterdam, the Netherlands

**Keywords:** Concept of fitness, natural selection

## Abstract

Fitness is the central concept in evolutionary theory. It measures a phenotype's ability to survive and reproduce. There are different ways to represent this measure: Malthusian fitness and Wrightian fitness. One can go back and forth between the two, but when we characterize model properties or interpret data, it can be important to distinguish between them. Here, we discuss a recent experiment to show how the interpretation changes if an alternative definition is used.

Fitness measures a phenotype's ability to survive and produce offspring that eventually become reproductive (Bürger 2000; Rousset 2004; Grafen 2007; Orr 2009). There are two common ways to define fitness. Malthusian fitness *m* refers to the exponential growth rate. With a population of size *N*(*t*) at time *t*, that implies 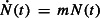. Wrightian fitness *w* is the average number of offspring, and is defined by *N*(*t* + 1) = *wN*(*t*). For Malthusian fitness, the solution is *N*(*t*) = exp (*mt*)*N*(0), and for Wrightian fitness, it is 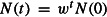. Time is naturally continuous if we use Malthusian fitness, while it is discrete for Wrightian fitness. Both models, however, lead to exponential growth in this most basic form. The relation between the two ways to define fitness is given by *m* = ln (*w*) (Crow and Kimura 1970; Bürger 2000; Orr 2009). For slow growth (corresponding to weak selection), a Taylor expansion of *m* = ln *w* for *w* ≍ 1 leads to *m*≍*w* − 1.

In experiments, one may want to measure fitness over a range of manipulations in order to draw inferences about its determinants. The relationship between Malthusian and Wrightian fitness, which is linear under weak selection, *m*≍*w* − 1, becomes nonlinear under stronger selection. In general, Malthusian fitness is the logarithm of Wrightian fitness, and therefore the difference between the two options is the difference between using log-transformed and untransformed data of Wrightian fitness. Also for the interpretation of the results, it can make a difference which of the two is used, which suggests that it is worth investigating the implications of either choice.

As an example, we consider a study describing an experimental microbial system (smith et al. 2010). When starved of amino acids, *Myxococcus xanthus* cells aggregate to form a fruiting body. A small portion of cells develop into stress-resistant spores, while the majority die. Some strains sporulate super efficiently, and are therefore referred to as “cheaters", while strains with normal sporulation efficiency are referred to as “cooperators". Cheater strains spread efficiently when rare, but do so poorly when in high abundance. In the experiment, the sporulation efficiency *σ* is used as fitness (smith et al. 2010). The sporulation efficiency is the ratio between the number of cells surviving as spores and the total number of cells, and corresponds to the Wrightian fitness.

The experiment was performed on agar, where the population is mixed, and what is manipulated is the initial frequency of cooperators. It turns out that the Wrightian fitness *σ* of both cheaters and cooperators is almost exponential in the frequency of cooperators. smith et al. (2010) argue that this strong nonlinearity calls for a generalization of Hamilton's classical rule (Hamilton 1964; van Veelen 2007). However, Malthusian fitness, that is ln *σ*, is almost linear in the frequency of cooperators. In this note, we focus on how the interpretation of such an experiment, that is the need to generalize Hamilton's rule, can be changed by adopting an alternative definition of fitness.

As a preparation, we explore what the implications would be if Malthusian fitness were considered and it happened to be linear in the frequency of cooperators. An example close to the experimental data of smith et al. (2010) is the case in which the Malthusian fitness of a cooperator 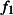 and a cheater 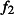 are


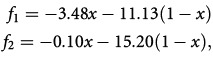
(1)

where *x* is the frequency of cooperators. Equivalently, in terms of game theory (Turner and Chao 1999; Nowak 2006), the payoff matrix *M* reads


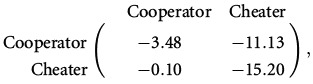
(2)

where 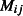 refers to the growth rate of *i* when the abundance of *j* in the population approaches 100%. In this case, the Malthusian fitness function is linear in the frequency of cooperators and the average fitness of the whole population, 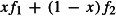, is quadratic. The average Malthusian fitness of the whole population reaches its maximum when the population consists of cooperators only.

Both Malthusian fitness functions are of the form 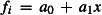. We can transform this to Wrightian fitness; 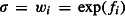. A similar approach has been proposed in Traulsen et al. (2008), because it is often easier to deal mathematically with such exponential functions. smith et al. (2010) proposed to perform a Taylor expansion of the Wrightian fitness function with respect to the frequency of cooperators to explore its nonlinearity. In the case of linear Malthusian fitness, we obtain for the Wrightian fitness


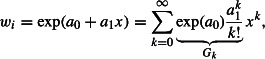
(3)

where 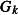 is the 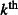 order of the Taylor coefficient of the Wrightian fitness function of a cheater or cooperator. The difference between two consecutive Taylor coefficients is


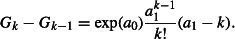
(4)

Thus, for exponential growth (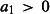), 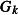 is increasing when *k* is smaller than 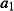 and decreasing when *k* is greater than 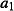. In other words, 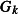 has a unique maximum around 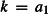. This unique maximum is a direct consequence of the assumption that Wrightian fitness is exponential and independent of the fitted values. The Taylor expansion of the Malthusian fitness is trivial in this case; if the Malthusian fitness is linear in the frequency of cooperators, the Taylor coefficients of the Malthusian fitness will all be zero, except for the first two. The Wrightian fitness function, on the other hand, will be nonlinear, and its Taylor coefficients will always come in the shape of a hump; they increase until 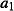 and decrease thereafter.

smith et al. (2010) find that the Wrightian fitness functions of both cheaters and cooperators are not exponential, but only almost exponential in the frequency of cooperators, see Fig. 1. Because they are close to exponential, the authors log transformed the data and performed an ordinary least squares fit on those transformed data. But because the fitness functions are not exactly exponential, they minimize the squared differences between the data and a quadratic function, 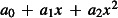, rather than between the data and a linear function, 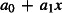. This results in estimates 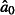, 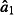 and 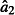, which translates into a Wrightian fitness function as follows: 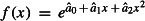. Finally, a Taylor expansion of this function is made, up to the 30th order, and a maximum with respect to the order of the Taylor coefficients is found. However, the calculations above and in the Appendix show that the shape of this curve is determined by the function chosen for the fit, and not by the data.

**Figure 1:**
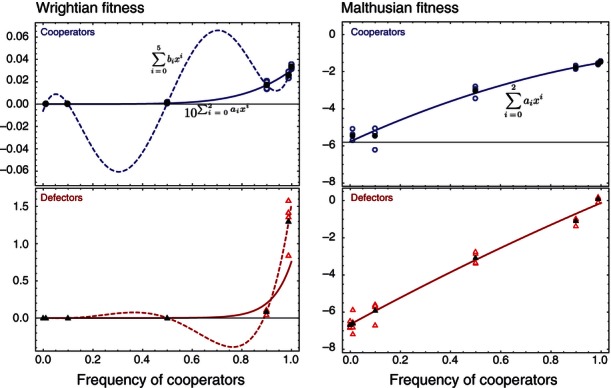
In smith et al. (2010), Wrightian fitness is employed. The Wrightian fitness from an experiment is log transformed, and a quadratic fit is performed. Then, a Taylor expansion of the function (solid curves in the left panels) is done, showing a maximum in the Taylor coefficients for defectors. In this case, there can be infinitely many nonvanishing Taylor coefficients. If the Wrightian fitness, however, is fitted directly by a polynomial, there are at most six nonvanishing Taylor coefficients (the dashed curves), as there are six distinct sample points with different frequencies of cooperators. In such a case, the result is dramatically different. Notably, even though there are infinitely many Taylor coefficients for exponential Wrightian fitness, it is still not a perfect fit in the sense that errors can actually be reduced even further. The dashed curve does that: it is a perfect fit that passes through all the means of the untransformed data points of the Wrightian fitnesses (left bottom panel). However, if the Malthusian fitness is used, (the right panels), there is no need to transform the data any longer, as it is almost “linear” already.

Why the final answer is expressed in terms of 30 Taylor coefficients is not immediately clear. Taylor expansions are local approximations useful for theoretical exercises, where a simpler expression is desired. For example, when the intensity of selection is small, complicated expressions can be linearized and become much easier to handle. A Taylor expansion in the present context suggests that we would like to approximate the Wrightian fitness function with a polynomial in the abundance of cooperators. That is remarkable, because one would expect (and we agree) that there were very good reasons why the functional form 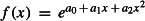 was chosen in the first place. But, if we were interested in a good match with a polynomial, we could achieve a perfect fit by going to polynomials directly with the untransformed data. Note that there are only six different frequencies of cooperators, which implies that we can choose a Wrightian fitness function that minimizes the squared differences with only six parameters. That can be done with any polynomial with a constant term and five additional nonzero Taylor coefficients. However, one may want to impose the constraint that these polynomials should be nonnegative. Overall, the procedure to first process the data with only three degrees of freedom, and then producing 30 Taylor coefficients seems to be questionable. Also, the calculation of the first 30 moments of a distribution with a support of only six points is problematic, but we acknowledge that the authors aim to prove a principle rather than propose a way to analyze such data. In the Appendix, we show that if the exponential quadratic term 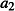 is sufficiently small compared with the linear one 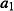, then the hump shape in the sequence of Taylor coefficients remains.

In this note, we point out that it can be important which kind of fitness is chosen. It has been noted that the deviation of weak selection may lead the fitness away from linearity (Van Dyken and Wade 2012). However, these authors do not discuss what type of fitness is addressed, but the work of smith et al. (2010) shows that this becomes an issue whenever selection is nonweak. In fact, if fitness effects are small, or the selection intensity is weak, then the difference between Wrightian fitness (close to 1) and Malthusian fitness (close to 0) does not matter. In this case, we have *m* ≍ *w* − 1. That implies that there is hardly any difference in linearity between a fitness effect in Wrightian terms and Malthusian terms. But if fitness effects are really large, as they are in the experiment of smith et al. (2010), then there can be an enormous difference. The goal of smith et al. (2010) is to generalize Hamilton's rule for a nonlinear fitness function, or to bridge the gap between the nonlinear fitness data and the previous theory (Van Dyken et al. 2011). Fitness, however, reduces to be linear as in equation (1), by replacing the Wrightian with the Malthusian fitness. This suggests that for this experiment, it seems unnecessary to use the generalized Hamilton's rule, if instead the Malthusian fitness is adopted. In other words, the Wrightian fitness approach calls for a generalization of Hamilton's rule, whereas the Malthusian fitness approach does not (or at least not in a drastic way, as Malthusian fitnesses are almost linear in the frequency of cooperators). However, there are of course cases in which fitness is neither exponential nor linear. Bacterial populations are growing continuously. Typically, generation times are short, suggesting that Malthusian fitness may be the better option (Lenski et al. 1991). Employing Malthusian fitness implicitly seems to suggest an exponential growth and death of the population. In addition, the development of *Myxococcus xanthus* consists also of a lag period and stationary regime (Kraemer et al. 2010). In all these cases, the log transformation from Wrightian to Malthusian fitness is also valid, that is, for shrinking populations or constant population size.

While we criticise these mathematical issues, we are convinced that smith et al. (2010) aim into the right direction: to incorporate the nonlinearities characteristic of biology into social evolution, we may have to extend and generalize the approach of inclusive fitness. It would be beautiful if such a generalization would ultimately include Hamilton's original rule as a special case in which nonlinearities vanish, as in the work of smith et al. (2010).

## Appendix

Here, we are addressing the expression for the  order Taylor coefficient  of the function  , and then investigate the monotonicity of . By using the analytical expression of the exponential function, making use of the binomial theorem, and rearranging the terms, we obtain

(A1)

Let *k* − *j* = *l* and *k* + *j* = *m*. Then we have

(A2)

where  is the set of all the odd integers ranging from 1 to *m*, if *m* is odd, and is the set of all the even integers ranging from 0 to *m*, if *m* is even.

Or, explicitly

(A3)

To investigate the monotonicity of , we only need to evaluate the difference between  and . Since there are different expressions of  for even *m* and odd *m*, we need to address  as well as . For , we have

(A4)

When  is sufficiently small compared to , the right side of equation A4 is determined by the term without , and the above difference can be approximated by

(A5)

Similarly, we have that  These are consistent with equation 4. Therefore, by the same arguments,  has a maximum around , provided the linear term  is positive and the quadratic term  is much smaller than .
